# The co-evolution of friend and help relationships and their different relationship formation and social influence

**DOI:** 10.1038/s41598-023-43346-w

**Published:** 2023-09-25

**Authors:** Huiyoung Shin

**Affiliations:** Department of Psychology, Jeonbuk National University, 14-3 Social Science Building, Deokjin-gu, Baekje-daero 567, Jeonju, 54896 Chonbuk Republic of Korea

**Keywords:** Health policy, Epidemiology

## Abstract

This study examined adolescents’ friend and help relationships to better understand their interrelated nature and the different characteristics of relationship formation and social influence in the behavioral and emotional dimensions of academic engagement. Multiplex networks of friends and helpers were collected (*N* = 542; *M*_age_ = 11.46; 20 classrooms) and analyzed using a multilevel Bayesian social network analysis (multilevel random-coefficients SAOM). The results showed that exchanging help played a role in the formation of friendships, and that friendships provided a relational context wherein help can be exchanged. Observable behavioral academic engagement played a more salient role in the formation of friend and help relationships, and highly engaged (in behavioral) adolescents were more often nominated as helpers. Both the behavioral and emotional dimensions of friends’ and helper’ academic engagement contributed to adolescents’ own behavioral and emotional engagement over time, but the social influence was more salient among friends compared to helpers. These results underscore that examining the dependencies among multiple networks and distinguishing between different dimensions of behavior and emotion are critical to elucidate the complex processes of relationship formation and social influence.

## Introduction

Academic engagement among adolescents has received much attention because it plays a crucial role in their future adjustment. It not only predicts their learning and achievement in school but also offers protection from risky behaviors such as dropout and delinquency^1^. Thus, many researchers have sought to identify the significant contributors to adolescents’ academic engagement, such as parents at home and teachers at school^2^. In recent years, increasing evidence has shown that peers are also an important socializing influence on adolescents’ academic behaviors. As adolescents spend more time with friends and peers, the features of their peer relationships become immensely influential on their own academic behaviors and attitudes^3^. Researchers have examined how and to what extent adolescents’ friends affect their academic engagement and indicated that individuals’ selection of friends and friends’ influence play a critical role^4^. Adolescents are more likely to seek out friends who are similar to them in terms of academic engagement (i.e., selection of friends), who then serve to socialize their academic engagement (i.e., influence of friends) over time (see^5^ for a review).

Although there has been extensive research showing that friend selection and influence contribute to changes in adolescents’ academic engagement, most prior research has focused on observable behavioral engagement such as school attendance or effortful behavior^4^. However, academic engagement is a multidimensional construct that includes both observable behavior (e.g., participation and absence of misconduct) and unobservable emotion (e.g., interest and enjoyment;^6^). Because prior research has focused on observable behavior without distinguishing between the different dimensions of behavior and emotion, the current understanding of peer effects on academic engagement is limited in scope. Further, most extant research on peer influence has primarily considered adolescents’ friends (e.g.,^7,8^). However, adolescents have diverse social interactions with their peers, and each peer relationship can have different implications for their psychological state as well as behavioral tendencies. It is therefore critical to specify and investigate the relationship processes beyond friends to better understand the unique roles of different peers.

Going beyond the focus on adolescents’ friends and their observable behaviors in prior research, the present study investigated early adolescents’ friends and helpers to better understand their interrelated nature as well as the different aspects of relationship formation and social influence in the behavioral and emotional dimensions of academic engagement. Considering help relationships in tandem with friendships will allow us to better understand the emergence and development of help relationships in adolescents’ peer contexts as well as any social influence that peer relations other than friends exert on adolescents’ academic engagement. Moreover, examining the different dimensions of academic engagement (i.e., observable behavior and unobservable emotion) will contribute to the current body of research by offering more insights into how each dimension of academic engagement is differentially predictive of peer relationship formation and likely to be uniquely influenced by peer characteristics^6^.

### Who becomes friends with whom and who helps whom?

The features and functions of peer relations could play important roles in relationship formation. Friendships are voluntary affiliations in which individuals frequently interact with each other, spend a lot of their time together, and like each other^9^. Intimate relationships and frequent interactions are generally contingent on similarities in behaviors because similarity enables individuals to connect with less effort and with shared feelings of understanding. Uncertainty reduction theory^10^ emphasizes that similarity between individuals lessens uncertainty and therefore increases interaction quality, which leads to relationship formation. Similarly, interpersonal attraction theory suggests that similarities between individuals increase positive affect, thus leading to more pleasurable interactions than those between dissimilar partners. Like uncertainty reduction theory, interpersonal attraction theory holds that positive and pleasurable interactions stimulate relationship formation between similar interaction partners^11^.

Thus, a set of theoretical frameworks emphasizes that similarity is a pervasive principle of relationship formation, and that positive interactions based on similarities stimulate intimate relationship formation, such as friendships. When individuals are similar in terms of their behaviors and attributes, they are more likely to offer mutual positive reinforcement, which in turn makes their relationships more rewarding and stable^5^. Adolescents’ congruent expectations about desired academic behavior could facilitate communication and trustworthiness as well as minimize conflict. It is therefore reasonable to expect that early adolescents are more likely to seek out friends who already possess similar behavioral proclivities or like-minded attitudes in terms of their academic characteristics.

Based on the similarity–attraction approach discussed above, help relationships can also be expected to emerge between individuals who have similar levels of academic engagement^12^. Individuals are thus expected to be attracted to peers who demonstrate a similar focus on academic effort^4^, because shared similarities in participation and interest in academic work ensure that their needs and goals can be more easily understood. Mutual understanding would make communication around academic challenges smoother and more efficient while also minimizing the threats of ridicule or rejection. Thus, academically engaged adolescents might try to seek out helpers who are also academically focused, because these relationships could provide safer emotional space to discuss their academic difficulties. However, the need to receive help from specific peers might only arise when those peers possess complementary characteristics that could provide academic benefits^13^. The help seeking–giving relationship is asymmetrical in nature in that the adolescents who need assistance turn to helpers for instructional needs such as information and advice about academic tasks as well as assistance with difficulties in academic problems^14^. Thus, adolescents who need help and want to improve their academic skills will be more likely to ask for help from peers who are more academically competent and display academic-oriented behaviors compared to themselves^15^.

Based on these reasoning and prior findings, we hypothesize that individuals who are similar in academic engagement will be more likely to nominate each other as friends and helpers. Moreover, given that help seeking–giving relationships are expected to emerge among individuals who possess complementary characteristics, we hypothesize that individuals’ academic engagement will be positively associated with being nominated as a helper (giving help). However, we also anticipate that the nature of individual attributes is likely to affect the selection of friends or helpers. Non-observable individual attributes such as beliefs or affective states are less likely to be critical in the initiation phase of relationship formation, because individuals need to be familiar before they can evaluate whether friends or helpers are similar in this respect^16^. On the contrary, readily observable individual attributes such as external behaviors are more likely to play a role in the selection of friends or helpers (e.g.,^17^). Because emotional engagement is largely non-observable, it is reasonable to expect that similarity in emotional engagement may not play a critical role in relationship selection. By contrast, because behavioral engagement is an indicator that is external and readily observable by peers, behavioral engagement among individuals can provide peers with a convenient sorting process to assess compatibility. Thus, we hypothesize that individuals who are similar in the behavioral dimension of academic engagement will be more likely to nominate each other as friends or helpers, whereas similarity in the emotional dimension of academic engagement between individuals will not contribute to the relationship selection.

### To What extent do friends and helpers influence adolescents’ academic engagement?

Once relationships are formed and maintained, the behavioral and emotional academic engagement of friends and helpers are presumed to contribute to adolescents’ own behavioral and emotional engagement. Social learning theory^18^ postulates that behaviors are socialized through processes such as modeling, reinforcement, and information exchange. Observing peers perform a particular academic task or express certain academic values and emotions can encourage individuals to engage in new academic behaviors or consider new viewpoints and inform individuals of the consequences of such behaviors and opinions^19^. Depending on these consequences, observing peers’ behaviors or emotions can bolster or lessen the likelihood that individuals will engage in such behaviors and emotions in the future. When focusing on academic work in class is discouraged or received negatively by peers, adolescents might be less likely to display such behaviors or emotions. By contrast, when such behaviors or emotions are positively received by peers, adolescents are expected to be more likely to display such behaviors or emotions^20^.

Both friends and helpers are expected to have substantial social influence on adolescents’ behavioral and emotional academic engagement over time due to their increased interactions with each other. The potential influence that friends have lies most clearly in frequent interactions based on intimacy. Adolescents spend most of their time with close friends whereby they share both academic and social experiences^21^; they develop their intimate relationships by committing to similar activities and behaviors. Close relationships could promote socialization toward increased similarities, as early adolescents strive to accommodate each other’s opinions, establish common grounds, and reach decisions through consensus^22^. Mutual reinforcement of specific behaviors and attitudes (e.g., via verbal encouragement, smiling, and nodding) is particularly present within intimate friendships^23^. Based on this view, it is expected that friends will influence each other’s behavioral and emotional dimensions of academic engagement over time.

By contrast, the potential influence of helpers lies in modeling and information sharing. When adolescents ask for help with their academic tasks, helpers provide not only immediate help but also valuable advice to alleviate their academic difficulties^24^. Helpers can share their own experiences that could facilitate learning as well as suggest positive emotions that could promote academic engagement. As early adolescents build supportive relationships with helpers, they have more opportunities to observe helpers’ behaviors and attend to helpers’ academic attitudes^25^. Through first appraising the norms and standards of helpers and then establishing or modifying behaviors and affective states that are appreciated by helpers^26^, adolescents may gradually adopt the academic involvement and interests of peers who help them (e.g.,^27^). Therefore, we hypothesized that adolescents will socialize the behavioral and emotional academic engagement of their friends and helpers, and that they will become more similar in terms of behavioral and emotional engagement to their friends and helpers over time.

### The co-evolution of friend and help relationships

Previous research has established the existence of an association between friend and help relationships, thus indicating that exchanging help can take place and develop in the relational context of friendships^28^. It is reasonable to assume that adolescents who experience academic challenges and need information or assistance would rely on friends for help because giving and receiving help is part of the expectations of friendships^29^. Friendships involve a need for mutuality and reciprocal exchanges, with increased emphasis on sharing, disclosure, and companionship^30^. As genuine mutual concern and caring are identified as central expectations of friendships, and as mutual intimacy can be bolstered through giving and receiving help, friends can be a reliable source of support and friendships are likely to become a salient context for helping interactions.

However, the associations between friend and help relationships are not necessarily unidirectional, as they can also be reciprocal. Friendships can provide a salient foundation for exchanging help, but giving and receiving help can also easily become a conduit for establishing new friendships^31^. Giving help to others entails spending time and effort, which can communicate affection and caring to the receivers. Further, asking for help from peers requires disclosure of academic shortcomings^32^. Because this disclosure could be a threat to a help-seeker’s self-esteem, seeking help conveys the message of willingness to share and disclose one’s weakness in front of helpers. Thus, asking for help can send helpers signals a desire for a close relationship and trustworthiness, which can lead to the formation of friendships^33,34^. Based on this, we hypothesized that existing friendships will contribute to the initiation of help relationship, and help relationships will increase the chance for friendship formation.

### The present study

The current study aimed to fill the existing gaps in the knowledge concerning the characteristics of different peer relationships and their implications in shaping individuals’ academic engagement by focusing on multiplex networks of friends and helpers. An additional aim was to elucidate the interrelated nature of friend and help relationships and examine how they co-evolve over time. To this end, we examined early adolescents’ friend and help networks as both dyadic and network processes, and we investigated relationship formation and social influence in relation to the behavioral and emotional dimensions of academic engagement.

To address our research questions, we used a short-term longitudinal design wherein two waves of data were collected over one school semester. We focused on early adolescence because this is a developmental period in which youth experience an increased desire to fit in with their peers. With the onset of puberty, early adolescents face increasing sensitivity to evaluations from others, and peer relations become their salient concerns^35^. In an effort to fit in, many early adolescents adjust their behaviors and attitudes to those of their peers to be appreciated positively by others they value^20^. We examined our research hypotheses using a multilevel Bayesian social network analysis, which is a multilevel extension of the SAOM of social network dynamics^36–38^. The SAOM was proposed for use in modeling network panel data^39^, and it was extended to a joint model for changing actor and network tie variables^40^ and also extended to a multiple networks model^41^. The multilevel random-coefficients SAOM incorporates random coefficients like the multilevel models. Thus, it estimates parameters across all classroom networks but allows for more variation in the estimated parameters between the classes. By using multiplex network data on early adolescents’ friends and helpers collected from multiple classrooms, we could use this analytic method to examine the direct effects of help relationships on friendships and vice versa, and to disentangle relationship selection and social influence by examining changes and stability in peer networks and individuals’ behavioral and emotional academic engagement with sufficient statistical power to account for between-class heterogeneity.

## Results

### Multilevel SAOM results

Tables 1 and 2 present a summary of the average changes in adolescents’ academic engagement and social networks from Wave 1 to 2. To model a SAOM with sufficient statistical power, a sufficient fraction of peer nominations should remain stable (i.e., Jaccard index). The Jaccard indices in our social networks were 42% for friends and 31% for helpers, which are values that are sufficiently high to use a SAOM^42^. Table 3 presents the multilevel random coefficients SAOM results for adolescents’ behavioral and emotional dimensions of academic engagement in multiplex networks of friends and helpers. The multilevel SAOM incorporates Bayesian inference estimation. Bayesian inference estimation assigns a prior probability distribution to the parameters, which is updated to a posterior probability drawing information from new data. We presented the estimated mean and across-classroom standard deviation for both randomly varying (i.e., posterior means μ and *sd* (μ)) and fixed (i.e., posterior means η and standard deviations *sd* (η)) parameters. We also provided Bayesian* p*-values (i.e., *p*_bayes_) of estimated parameters, which represent the percentile below zero in the posterior distribution. Bayesian *p*-values of ≥ 0.975 and ≤ 0.025 indicate a high chance that the alternate hypothesis is true (i.e., a parameter being positive for *p*-values of ≥ 0.975 and negative for *p*-values of ≤ 0.025).

**Table 1:** Changes in early adolescents’ behavioral and emotional academic engagement.

	Wave 1–Wave 2		
Behavioral academic engagement			
Mean (*SD*)	3.37 (1.01)		3.36 (0.91)
Fraction increased		22.7%	
Fraction decreased		24.5%	
Fraction stable		52.8%	
Emotional academic engagement			
Mean (*SD*)	3.33 (1.02)		3.28 (0.91)
Fraction increased		24.7%	
Fraction decreased		25.9%	
Fraction stable		49.4%	
*N* of classes	20		20
*N* of individuals	542		514

**Table 2:** Changes in early adolescents’ social networks of friends and helpers.

	Friends		Helpers	
	Wave 1	Wave 2	Wave 1	Wave 2
Social network indicators				
Average (range) class size	27 (23–31)	27 (23–31)	27 (23–31)	27 (23–31)
Density	0.15_a_	0.13_a_	0.07_b_	0.06_b_
Reciprocity	0.87	0.89	0.87	0.88
Transitivity	0.53_a_	0.54_a_	0.25_b_	0.26_b_
Centrality (indegree)	0.15_b_	0.15_b_	0.32_a_	0.32_a_
Centrality (outdegree)	0.19_a_	0.20_a_	0.05_b_	0.06_b_
Social network changes	Wave 1–Wave 2		Wave 1–Wave 2	
Average *n* of nominations	3.71		1.69	
Average *n* of ties dissolved	36.0 (25.2%)		28.3 (40%)	
Average *n* of ties emerged	51.8 (35.6%)		24.0 (33.9%)	
Average *n* of ties maintained	55.8 (39.2%)		18.6 (26.1%)	
Hamming distance (change)	153.4		97.24	
Jaccard index (stability)	0.42		0.31	

Density is the proportion of actual nominations relative to the total possible nominations; Reciprocity is the proportion of mutual ties; Transitivity is the proportion of tie configurations that could become cohesive peer groups; Centrality is the proportion of all received or sent nominations relative to the total possible nominations; Hamming distance is the amount of tie changes from the beginning to the end of the time point; Jaccard index is the fraction of stable ties relative to all new, lost, and stable ties; Means in the same row that do not share subscripts differ at *p* < .001 in the independent *t* test between friend and help network structure for wave 1 and 2, respectively.

**Table 3:** Multilevel SAOM estimates in multiplex networks of friends and helpers.

Effect	Friends					Helpers				
	par	psd	*p*	95% CI		par	psd	*p*	95% CI	
Friend network structural effects										
Rate of network change	**7.25**	0.41				**6.93**	0.49			
Outdegree (density)	**− 1.94**	0.13	< .01	**− **2.21	**− **1.69	**− 1.24**	0.11	< .01	**− **1.46	**− **1.02
Reciprocity	**1.39**	0.09	> .99	1.22	1.58	**1.63**	0.09	> .99	1.45	1.80
Transitive triplets	**0.60**	0.04	> .99	0.52	0.68	**0.68**	0.04	> .99	0.59	0.76
Transitive reciprocated triplets	**− 0.34**	0.05	< .01	**− **0.44	**− **0.23	**− 0.39**	0.05	< .01	**− **0.49	**− **0.29
Indegree popularity	**− 0.10**	0.03	< .01	**− **0.12	**− **0.03	**− 0.11**	0.03	< .01	**− **0.17	**− **0.06
Outdegree activity	**− 0.08**	0.02	< .01	**− **0.12	**− **0.03	**− 0.09**	0.02	< .01	**− **0.14	**− **0.05
Help network structural effects										
Rate of network change	**4.15**	0.30				**5.18**	0.44			
Outdegree (density)	**− 1.02**	0.16	< .01	**− **1.30	**− **0.69	**− 1.96**	0.20	< .01	**− **2.35	**− **1.59
Reciprocity	**1.03**	0.12	> .99	0.80	1.26	**0.32**	0.11	> .99	0.08	0.56
Transitive triplets	**0.92**	0.08	> .99	0.77	1.07	**0.58**	0.08	> .99	0.40	0.75
Transitive reciprocated triplets	**− 0.45**	0.16	< .01	**− **0.78	**− **0.17	**− **0.14	0.17	.21	**− **0.57	0.18
Indegree popularity	**0.13**	0.01	> .99	0.11	0.15	**0.16**	0.02	> .99	0.13	0.20
Outdegree activity	**− 0.45**	0.05	< .01	**− **0.56	**− **0.36	**− 0.40**	0.05	< .01	**− **0.50	**− **0.30
Cross-network effects										
Helper to friend	**1.45**	0.10	> .99	1.24	1.68					
Friend to helper						**1.58**	0.09	> .99	1.39	1.75
Network selection effects										
^a^Gender alter	**− **0.05	0.07	.23	**− **0.18	0.09	**− **0.01	0.08	.47	**− **0.17	0.14
^a^Gender ego	0.08	0.07	.85	**− **0.06	0.23	**− **0.08	0.11	.24	**− **0.30	0.15
Same gender	**0.63**	0.08	> .99	0.48	0.79	**0.40**	0.10	> .99	0.24	0.58
Behavioral academic engagement alter	**− 0.11**	0.03	< .01	**− **0.17	**− **0.03	**0.25**	0.05	> .99	0.15	0.34
Behavioral academic engagement ego	**0.12**	0.04	> .99	0.05	0.19	0.02	0.06	.60	**− **0.10	0.14
Similar behavioral academic engagement	**0.33**	0.13	> .99	0.07	0.59	**0.36**	0.17	.98	0.03	0.72
Emotional academic engagement alter	**− 0.14**	0.04	< .01	**− **0.19	**− **0.04	0.02	0.04	.72	**− **0.06	0.11
Emotional academic engagement ego	**− 0.08**	0.04	.04	**− **0.12	0.04	**− **0.04	0.07	.34	**− **0.19	0.11
Similar academic emotional engagement	0.20	0.13	.93	**− **0.06	0.46	0.05	0.19	.39	**− **0.47	0.32
Behavior effects: behavioral engagement										
Rate of change	**1.43**	0.19				**1.50**	0.19			
Linear shape	**− **0.18	0.17	.14	**− **0.53	0.14	**− **0.48	0.26	.03	**− **0.98	0.03
Quadratic shape	**− 0.15**	0.06	.01	**− **0.26	0.04	**− 0.26**	0.06	< .01	**− **0.38	**− **0.13
Effect from friend indegree popularity	0.06	0.04	.92	**− **0.02	0.14	**0.19**	0.04	> .99	0.11	0.27
Effect from friend outdegree activity	**− **0.02	0.04	.34	**− **0.09	0.07	**− **0.05	0.29	.36	**− **0.34	0.22
Average similarity	**1.81**	0.62	> .99	0.65	3.08	**2.53**	0.74	> .99	1.20	4.17
Behavior effects: emotional engagement										
Rate of change	**1.44**	0.15				**1.45**	0.15			
Linear shape	**− 0.47**	0.18	< .01	**− **0.82	**− **0.14	**− 0.72**	0.24	< .01	**− **1.17	**− **0.28
Quadratic shape	**− 0.14**	0.07	.04	**− **0.28	0.01	**− 0.22**	0.07	< .01	**− **0.34	**− **0.06
Effect from friend indegree popularity	**− **0.04	0.04	.18	**− **0.13	0.05	**0.10**	0.03	> .99	0.04	0.15
Effect from friend outdegree activity	**0.16**	0.04	> .99	0.06	0.25	**0.28**	0.15	> .99	0.02	0.56
Average similarity	**1.42**	0.60	> .99	0.23	2.67	1.06	0.59	.97	**− **0.01	2.30

Par. = Posterior means η and standard deviations *sd* (η) for fixed parameters, and posterior means μ and *sd* (μ) for random parameters (i.e., network structural effects and same gender effect for friend networks). Bayesian *p*-values indicate the percentile below zero in the posterior distribution. Bayesian *p*-values of ≥ 0.975 and ≤ .025 indicate a high posterior chance that the alternate hypothesis is true. The significant effects are presented in bold.

#### Who becomes friends with whom and who helps whom?

We found evidence for the similarity selection effect being positive for the behavioral dimension of academic engagement for both friends (*η* = 0.33, SD = 0.13, *p* > 0.99) and helpers (*η* = 0.36, SD = 0.17, *p* = 0.98). For the emotional dimension of academic engagement, we found weak evidence for the similarity selection effect for friends (*η* = 0.20, SD = 0.13, *p* = 0.93). In terms of receiving and giving peer nominations of friendship and help, we found negative alter effects of both behavioral (*η* = − 0.11, SD = 0.03, *p* < 0.01) and emotional (*η* = − 0.14, SD = 0.04, *p* < 0.01) academic engagement for friends, while we found positive alter effects of behavioral academic engagement (*η* = 0.25, SD = 0.05, *p* > 0.99) for helpers. Both friend and help relationships were more likely to be formed with peers of the same gender than different, as indicated by the same gender selection effects for both friends (*μ* = 0.63, SD = 0.08, *p* > 0.99) and helpers (*μ* = 0.40, SD = 0.10, *p* > 0.99).

#### To what extent do friends and helpers influence adolescents’ academic engagement?

We found evidence for the average similarity (social influence) effect being positive for both behavioral (*η* = 1.81, SD = 0.62, *p* > 0.99) and emotional (*η* = 1.42, SD = 0.60, *p* > 0.99) academic engagement in friend networks. In terms of help networks, we found evidence for the average similarity effects being positive for behavioral (*η* = 2.53, SD = 0.74, *p* > 0.99) academic engagement and weak evidence for emotional (*η* = 1.06, SD = 0.59, *p* = 0.97) academic engagement. These results held after controlling for the linear and quadratic tendencies of behavioral and emotional academic engagement as well as the effects of covariates (indegree popularity and outdegree activity). The positive indegree popularity effects in help networks were reflected in the finding that individuals who received many help nominations (gave help) displayed higher behavioral (*η* = 0.19, SD = 0.04, *p* > 0.99) and emotional (*η* = 0.10, SD = 0.03, *p* > 0.99) engagement. However, this pattern was not found in friend networks, indicating that the number of friendship nominations was not associated with the levels of either behavioral or emotional engagement.

#### The co-evolution of friend and help relationships

Regarding the cross-network effects, we found evidence for the helper to friend (*η* = 1.45, SD = 0.10, *p* > 0.99) and friend to helper (*η* = 1.58, SD = 0.09, *p* > 0.99) effects, thus indicating that help seeking-giving relationships increase the formation of friendships, and that existing friendships promote the formation of help relationships.

#### The network structural effects

The social networks of friends and helpers showed both similarities and differences in terms of their network structural features. The reciprocity effect was positive (*μ* = 1.39, SD = 0.09, *p* > 0.99; *μ* = 1.03, SD = 0.12, *p* > 0.99) for both social networks, indicating that individuals tend toward mutual relationships. Moreover, the positive transitivity triplets (*μ* = 0.60, SD = 0.04, *p* > 0.99; *μ* = 0.92, SD = 0.08, *p* > 0.99) and negative reciprocated transitivity triplets (*μ* = − 0.34, SD = 0.05, *p* < 0.01; *μ* = − 0.45, SD = 0.16, *p* < 0.01) effects indicate that individuals tend to form triadic relationships (e.g., friends of friends become my friends) and cluster in groups. The indegree popularity effect was negative for friend networks (*μ* = − 0.10, SD = 0.03, *p* < 0.01), while it was positive for help networks (*μ* = 0.13, SD = 0.01, *p* > 0.99), thus indicating that individuals ask for help from certain peers whom others often ask for help as well. The outdegree activity effect was negative for both social networks (*μ* = − 0.08, SD = 0.02, *p* < 0.01; *μ* = − 0.45, SD = 0.05, *p* < 0.01), indicating that individuals who nominate many peers as their friends and helpers tend not to form additional relationships and maintain existing ones.

**Figure 1:**
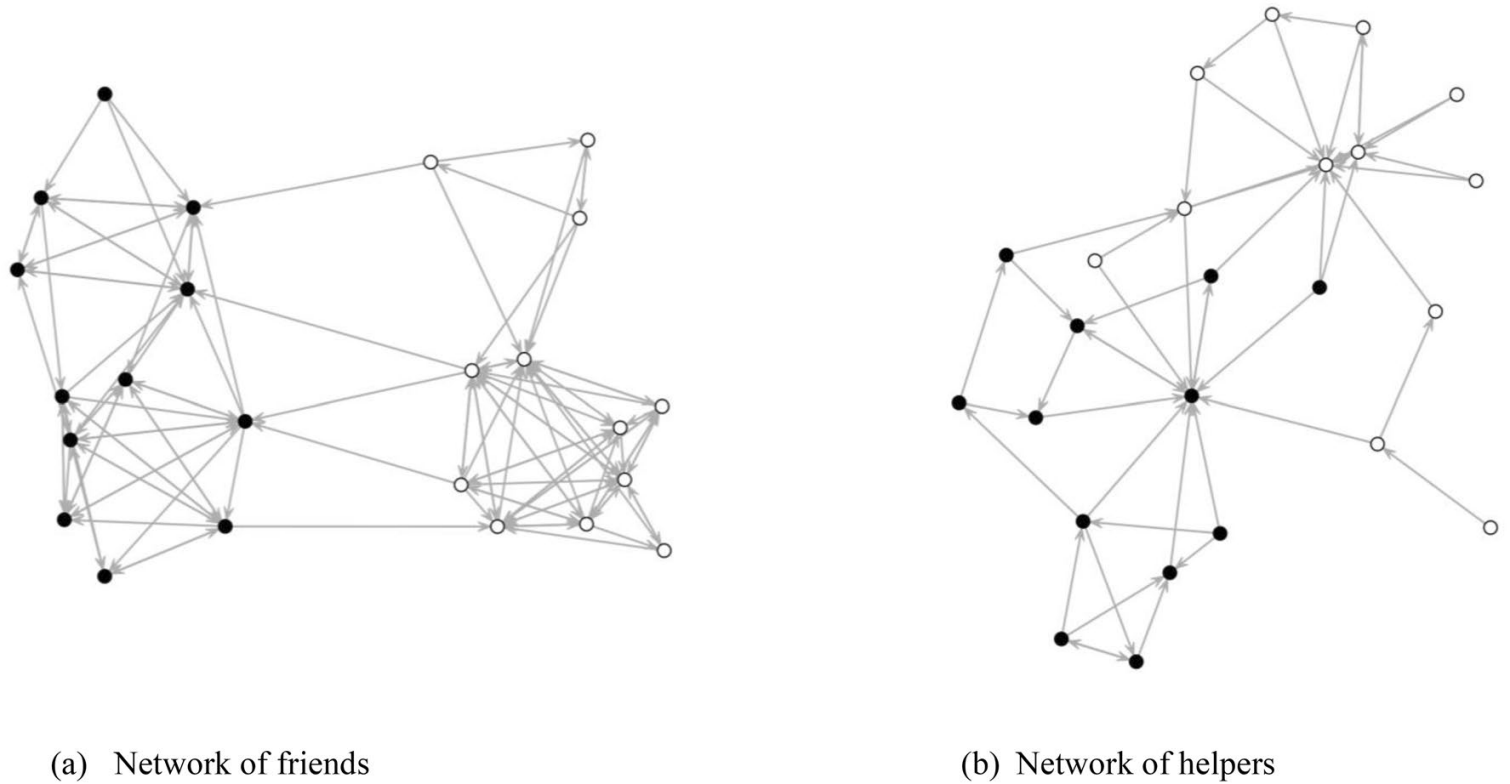
The social networks of friends and helpers. Social ties (arrows) are based on directed nominations between individuals (nods). The black nods are female, and white nodes are male.

These network structural features are reflected in Fig. 1, which graphically displays the sociograms of the friends and helpers in one classroom we investigated. This classroom is typical in the sense that it reflects the general network structural statistics of friend and help networks. The figure provides a graphical representation of the differences between two networks. In tandem with the descriptive statistics, these effects indicate that adolescents tend to reciprocate peer relations, keep their social networks closed, and form clusters in their friend and help networks. However, friend networks were on average twice as dense and transitive as help networks, and centralization of received nominations was more apparent in the help networks than it was in the friend networks, reflecting that help nominations were directed to a few individuals who could provide assistance to other peers.

## Discussion

Based on prior research evidence reporting on the dependencies between friend and help relationships^28,34^, we expected that friendships would function as an important context for seeking and giving help, and that exchanging help would contribute to friendship formation. In line with this expectation, we found that friendships indeed contributed to help exchange interactions, and that seeking and giving help had a direct impact on the formation of friendships. This result indicates how mutuality and reciprocal exchanges based on intimacy, sharing, and companionship can play an important role in adolescents’ willingness to seek and provide help. Given that friendships are characterized as symmetrical reciprocity, which refers to mutual acceptance and regard^43^, genuine mutual concern and caring can function as an encouraging context for exchanging help. The results also illustrate how helping interactions can increase the chance for new friendship formation. As giving help communicates affection and caring and as seeking help signals the potential for a close and trustworthy relationship, helping interactions may help establish new friendships^31^.

In addition to this interrelated nature of friend and help relationships, our primary aim was to investigate if there are differences in the characteristics of relationship formation and social influence between networks of friends and helpers. From the perspective that similarity between individuals increases interaction quality and positive affect and stimulates relationship formation^10^, we hypothesized that individuals who were similar in academic engagement would nominate each other as friends and helpers more often. In line with our hypothesis, we found that adolescents tended to select peers who were similar to them in academic engagement as friends and helpers. In general, adolescents tended to seek out friends and helpers who were similarly interested in academic work. Mutual understanding and similar levels of interest in academic work would offer mutual positive reinforcement and congruent expectations about academic behavior could facilitate communication and trustworthiness and minimize conflict, which can reasonably lead to friendship formation. Such aspects could also be critical in initiating help relationships. because seeking help from peers could have social repercussions, as it requires disclosure of academic shortcomings^32^. Because this disclosure could be a threat to help-seekers’ self-esteem and hinder positive peer evaluations from others^44^, adolescents would be hesitant to exhibit their academic weakness in front of peers unless they find helpers who are similarly interested in academic work, as it provides safer emotional space to discuss their academic challenges.

However, confirming prior study findings that non-observable individual attributes such as affective states are less critical in the initiation phase of relationship formation^16^, similarity in behavioral academic engagement was found to more strongly contribute to the selection of friends and helpers. Students can easily observe and quantify their peers’ behavioral academic engagement, such as paying attention and raising their hands; hence, behavioral engagement could provide peers with a convenient sorting process to assess compatibility. By contrast, emotional academic engagement is less easily observed and evaluated by peers, and accurate assessments require spending more time with peers; thus, individuals’ emotional academic engagement could play a lesser role in relationship formation^45^.

Further, individuals who were behaviorally focused on and emotionally interested in academic work tended to receive many help nominations (give help to other peers). Thus, adolescents who need help and want to improve their academic skills seem to ask for help from peers who are more academically competent and who display more academic-oriented behaviors compared to themselves^15^. Because academic engagement is positively associated with achievement and cognitive capacities^46^, highly engaged adolescents could be considered as ideal help providers; individuals were therefore more likely to approach these engaged youth to seek help with their academic difficulties. Interestingly, such academically engaged individuals received fewer friendship nominations from peers. This pattern of results reflects a concerning trend of declining academic motivation and engagement among adolescents^47^. It indicates that many adolescents do not focus on academic work, and thus, do not consider academically engaged peers as attractive to become friends with. It further suggests that they can experience a negative cycle by forming friendships with similarly disengaged peers.

Once relationships are formed and maintained, academic engagement of adolescents’ friends and helpers contributed to their own academic engagement. Increased time spent and interactions with friends and helpers translated to more closely aligned academic effort and interest over time. Frequent interactions based on intimacy and close mutual relationships could promote socialization toward increasing similarities, and mutual reinforcement via verbal encouragement, smiling, or nodding might bolster individuals’ engagement in specific academic behavior^23^. Further, observing peers perform a particular academic task or express certain academic values and emotions could provide individuals with opportunities to appraise their peers’ norms and standards as well as modify their own affective states^26^, thus leading youth to gradually adopt the academic (dis)involvement and (dis)interests of their peers.

However, the magnitude of social influence differed by the type of peer relationships. Whereas the social influence was similarly salient for behavioral and emotional academic engagement among friends, the social influence was only salient for behavioral academic engagement among helpers. This is in line with prior evidence indicating that strong social ties shaped within dense and cohesive social networks, such as between friends, are more powerful in transmission of behaviors^48^. Individuals’ affective states, beliefs, and behaviors are socialized through complex contagion processes^49^; that is, adopting certain behaviors or affective states is extremely difficult and requires multiple instances of social reinforcement. Because the aspects of academic engagement, such as paying attention, enjoying academic works, and trying hard in school, are costly and require sustained effort, dense social networks with more clustering are advantageous in providing multiple instances of social reinforcement that could significantly increase the likelihood that adolescents will adopt the behaviors and affective states over time. Furthermore, strong interpersonal affect, such as intimacy, can strengthen the social influence of behaviors and affects^50^, and close relationships and frequent interactions with friends could amplify the effects of dense and clustered social networks for promoting individuals’ academic engagement.

Although the social influence of helpers was comparatively weaker than that of friends, it should be noted that helpers also contributed to individuals’ academic engagement. Early adolescents with helpers who work hard on schoolwork and are invested in class were also more likely to display similar academic behaviors over time. The current findings in relation to friend networks indicated a magnified similarity selection and social influence when adolescents’ academic engagement was low, which is consistent with prior research findings showing that undesirable attributes are more vulnerable to social influence^51^. Given these findings, assembling distractive peers within the same setting could reinforce and worsen adolescents’ distractive behaviors^52^. Given the positive qualities of helpers (e.g., helpers displayed higher academic engagement) and their social influence in individuals’ academic engagement, researchers and practitioners should attend to adolescents’ help relationships to facilitate academically supportive peer social dynamics in the classroom. Given our findings demonstrating that friend and help relationships co-evolve over time, it is particularly important to foster help relationships in the informal peer interactions and motivate academically less engaged individuals to create social connections with highly engaged peers. Coaching educators to target helpers or other influential peers as agents of change could also promote a positive peer social influence on adolescents’ academic engagement^53^.

This study has several limitations that we need to acknowledge. First, our measure of social ties does not account for variations in relationship quality or strength. The strength of a social tie is a combination of the amount of time spent together, emotional intensity, and mutual confiding, as well as the reciprocal services that characterize the relationship^54^. However, we treated each social tie as equivalent in our analyses. Future research considering the strength of each social tie could provide valuable information about how psychological processes or observable behaviors are differentially influenced by the quality of social relations. Second, we only assessed adolescents’ relationships and their academic engagement at two time points, which could not reflect many of the changes that are likely to happen over the years. Future work that follows the same cohort of early adolescents across multiple years could help elucidate how different relationships around academic engagement change throughout adolescence against the backdrop of changing social contexts.

## Methods

### Participants and procedures

The data examined in this study was collected from a longitudinal project on early adolescents’ peer networks and school adjustment; the project was approved by the Institutional Review Board of the Oklahoma State University and all of the procedures were performed in accordance with the relevant guidelines and regulations. Four public elementary schools located in urban areas in South Korea agreed to participate in the project. All schools were in the same district, which serves a sizable proportion of middle-income families. The demographics, structures, and sizes of all schools and classrooms examined were comparable to each other. Elementary schools in South Korea contain grades 1–6, and students remain in a classroom with one teacher and the same peers for the entire day. We administered the surveys to students at the beginning of the academic semester in August for Wave 1 and at the end of the semester in December for Wave 2. Surveys were administered to participants in the classrooms, and informed consent forms were received from students, parents, and teachers prior to beginning the survey.

The project sample was comprised of students attending one of 26 classrooms in fifth and sixth grade in four schools. In both Waves 1 and 2, students nominated their peers (friends and helpers) and self-reported on the behavioral and emotional dimensions of academic engagement. Three classrooms were excluded from the sample because they did not participate in Wave 2. Another three classrooms were excluded from the final analyses because their model fit was below the customary value (for details see Appendix 1). The final sample (*M*_age_ = 11.46; *N* = 542 in Wave 1 and 514 in Wave 2) was about half female (52% in Waves 1 and 2) and ethnically homogeneous (more than 99% of students were South Korean). The participation rate was 97%, and the attrition from Wave 1 to 2 was 2.2%. We conducted attrition analyses to examine if there were differences between youth who completed both assessments and those who only completed the first assessment. The students who were only present in the first assessment were not significantly different from the students who participated in both assessments in their levels of behavioral and emotional academic engagement (*t* [540] = 0.22–0.62, *p* = 0.17–0.49).

### Measures

#### Social network

Social networks of friends (“who are your close friends that you hang around with and talk to the most?”) and helpers (“who do you most often ask for help with your academic difficulties?”) within classrooms were assessed using a peer nomination procedure. Participants were provided with a list of their classmates within the survey, then instructed to nominate an unlimited number of peers regardless of gender. Because this study focused on students' friend and help networks within classrooms across the school year and because students stay in a classroom with the same peers for the most part of the day, we only considered the social ties observed within classrooms, and not the social ties across different classes and schools. Based on peer nominations, we constructed *n* × *n* adjacency matrices for friends and helpers within classrooms, where *n* is the total number of classmates, with x_*ij*_ = 1 when there is a social tie from individual *i* to individual *j* and *x*_*ij*_ = 0 when there is no such social tie. There was some turnover from Wave 1 to Wave 2 because some students moved to other schools, so we analyzed the social networks of 542 participants in Wave 1 and their social ties that joined or left the peer networks in Wave 2 by coding the missing values as structural zeros^55^.

#### Academic engagement

We used the Rochester Assessment of Intellectual and Social Engagement (see^56^) to assess two dimensions of academic engagement: behavior and emotion. Behavioral engagement (three items) indicates the extent to which students pay attention to and participate in class, and emotional engagement (three items) indicates the extent to which students enjoy learning and are interested in school materials^57^. Sample items include “pay attention in class” and “enjoy learning new things in class”, and the scale was reliable in the current sample (α for behavioral engagement = 0.90 and 0.87 for Waves 1 and 2, respectively; α for emotional engagement = 0.89 for both Waves 1 and 2). All items were rated on a five-point Likert scale, with response anchors ranging from 1 to 5. The average score of the items was computed to form a composite scale for each dimension, with higher scores indicating of higher levels of behavioral and emotional academic engagement.

### Analytic strategy

#### Multilevel Bayesian social network analyses

To have sufficient statistical power and to account for potential between-group variation, multiplex classroom networks of friends and helpers were analyzed using multilevel Bayesian social network analysis (multilevel random-coefficients stochastic actor-oriented models; multilevel SAOM). Multilevel SAOM analyses rely on a Bayesian estimation of one or multiple groups when they have the same number of waves and the same model specification. We first estimated the multi-group models and performed the effect-wise joint significance tests to check the between-group variability of the effects. Most effects corresponding to the research hypotheses (i.e., cross-network effects, relationship formation and social influence effects) did not show significant variations across classes. Significant between-group variability was found in network structural effects of friend networks (e.g., reciprocity, transitivity, popularity) and same gender effects. Thus, parameters corresponding to the research questions were assumed to be constant across different classes to gain statistical power, while effects that showed significant variations across classes were allowed to vary randomly between classes. Bayesian inference estimation assigns a prior probability distribution to the parameters, which is updated to a posterior probability drawing information from new data. The estimates are derived from the iterative simulations using Markov Chain Monte Carlo algorithms^58^. Missing data (i.e., less than 3%) were handled in the SAOM, which allows for some missing data on network variables, covariates, and behavior variables. In a network simulation study, less than 10% of missing data do not provide estimation bias^55^. Reliable estimates are assessed with good convergence statistics of the estimation algorithm as indicated by convergence *t*-statistics for deviations from targets^38^. When the convergence* t*-statistics for all parameter estimates are less than 0.1 in absolute value, and the overall maximum convergence ratio is less than 0.25, convergence can be considered adequate^59^. All final models converged based on standard convergence assessments. We checked both the overall maximum convergence ratio and the goodness of fit. The goodness of fit trace plots based on method of moments are provided in the supplementary material (see Appendix 1).

#### Model specification

Analyses yield parameter estimates related to rate (rate of change between time points) and objective functions (the primary determinant of the probabilities of changes). Objective functions can be related to network (network structural, cross-network, and network selection effects) and behavior (behavior tendencies and influence effects) dynamics. We described the key aspects of what the models specified and estimated in the following. We included conceptual explanations and graphical representation of different effects in more detail in the supplementary material (see Appendix 2).

##### Network structural effects

To investigate the network characteristics, we included outdegree, reciprocity, transitive triplets, reciprocated transitive triplets, indegree popularity, and outdegree activity as endogenous network effects. *Outdegree* reflects individuals’ tendency to nominate others as friends or helpers. *Reciprocity* reflects individuals’ tendency to form mutual relations. *Transitive triplets* and *reciprocated transitive triplets* reflect individuals’ tendency to form transitive closure and its iteration with reciprocity^60^. *Indegree popularity* reflects the tendency of individuals who already receive many nominations to attract additional nominations. *Outdegree activity* reflects the tendency of individuals with high tendencies to nominate others as friends or helpers to make additional nominations.

##### Cross-network effects

To investigate the effects of one relationship on the other relationship, we included the effects of help on friendship (*helper to friend*) as well as the effects of friendship on help relationship (*helper to friend*).

##### Network selection effects

To investigate relationship selection based on behavioral and emotional dimensions of academic engagement, we included the effects of behavioral and emotional academic engagement on friendship and help nominations that are received (*alter effects*) and given (*ego effects*), and selecting similar others based on the levels of engagement (*similar selection*). Further, *ego* and *alter,* and *similar selection* were included for gender to control for the effects of gender on the number of nominations given and received, and the selection of similar peers based on gender.

##### Behavioral tendencies and covariate effects

We controlled for behavioral tendencies in behavioral and emotional academic engagement by adding effects indicating general tendency (*linear shape;* overall tendency) and dispersion (*quadratic shape*). We also included effects from *indegree popularity* and *outdegree activity* to control for the potential impacts of an individual’s popularity and activity on the levels of the behavioral and emotional dimensions of academic engagement.

##### Behavior social influence effects

To investigate the social influence of friends and helpers on the behavioral and emotional dimensions of academic engagement, we included an *average similarity effect*. This effect estimated whether individuals changed their levels of behavioral and emotional academic engagement to resemble their friends’ and helpers’ behavioral and emotional academic engagement.

##### Direction of selection and influence effects

Based on parameter estimates, we calculated *ego*-*alter* (nomination giver-receiver) selection and influence effects to fully explain the direction of significant effects (e.g., whether high-engaging and low-engaging individuals differ in terms of their preferences for high-engaging and low-engaging friends or helpers). The *ego*-*alter* selection and influence tables are provided in the supplementary material (see Appendix 3).

### Ethical approval

The studies involving human participants were reviewed and approved by the Institutional Review Board of the Oklahoma State University and all of the procedures were performed in accordance with the relevant guidelines and regulations.

### Informed consent

Informed consent forms were received from students, parents, and teachers prior to beginning the survey.

### Supplementary Information

Supplementary Information.

## Data Availability

Due to a privacy protection agreed with the Ethics commission, the full data set is not released publicly. Anonymized parts of the dataset are available upon request.
