# Supplementary material for: The co-evolution of friend and help relationships and their different relationship formation and social influence
Source: Sci Rep. 2023 Sep 25;13:15974. doi: 10.1038/s41598-023-43346-w (PMC10519936; doi:10.1038/s41598-023-43346-w)
Supplement: Supplementary file 1 — Supplementary Information. [file 41598_2023_43346_MOESM1_ESM.pdf]

Supporting Information

Appendix 1 shows the goodness of fit results of auxiliary statistics, including indegree distribution (Figure S1), outdegree distribution (Figure S2), triad census (Figure S3), geodesic distance (Figure S4), and behavioral (Figure S5) and emotional academic engagement (Figure S6), based on generalized method of moments.

Appendix 2 summarizes conceptual explanations and graphical representation of the basic parameters used in the SAOM model (Table S1).

Appendix 3 shows the *ego-alter* selection (Table S2–S3) and influence tables (Table S4–S5).

Appendix 1

Goodness of fit. We evaluated the goodness of fit with auxiliary statistics. Four auxiliary network statistics (i.e., indegree distribution, outdegree distribution, triad census, and geodesic distance) and two auxiliary behavior statistics (i.e., behavioral and emotional academic engagement) were assessed. The auxiliary network statistics indicate how well relationship patterns in the networks are reflected with the included model effects. For each auxiliary statistic, the differences between the simulated values in the model and the observed values in the data are compared with the Mahalonobis distance. The results of goodness of fit can be plotted with violin plots, which can be used to assess if too many or few effects are simulated in the model comparing to the observed values in the data. The red lines indicate the observed values and the boxplots and violin plots indicate the distribution of the simulated values. The *p*-values for the goodness of fit can be also calculated with respect to the distance from the center of the cloud, taking into account the correlations and variances of the auxiliary statistics. The customary value of $p = .05$ can be used as a threshold checking whether the goodness of fit is adequate [59]. The results of the goodness of fit are provided figuratively below with the corresponding *p*-values. Overall, the goodness of fit of all auxiliary statistics were good. The simulated values in the model did not depart far from the observed values in the data, and the *p*-values for all auxiliary statistics were above .10 in most classes. Based on the assessment, three classrooms were excluded in the final analyses because their goodness of fit was below the customary value of .05. The final results with and without three classrooms were comparable in the pattern of the parameter estimates. However, the results without three classrooms provided smaller SD.

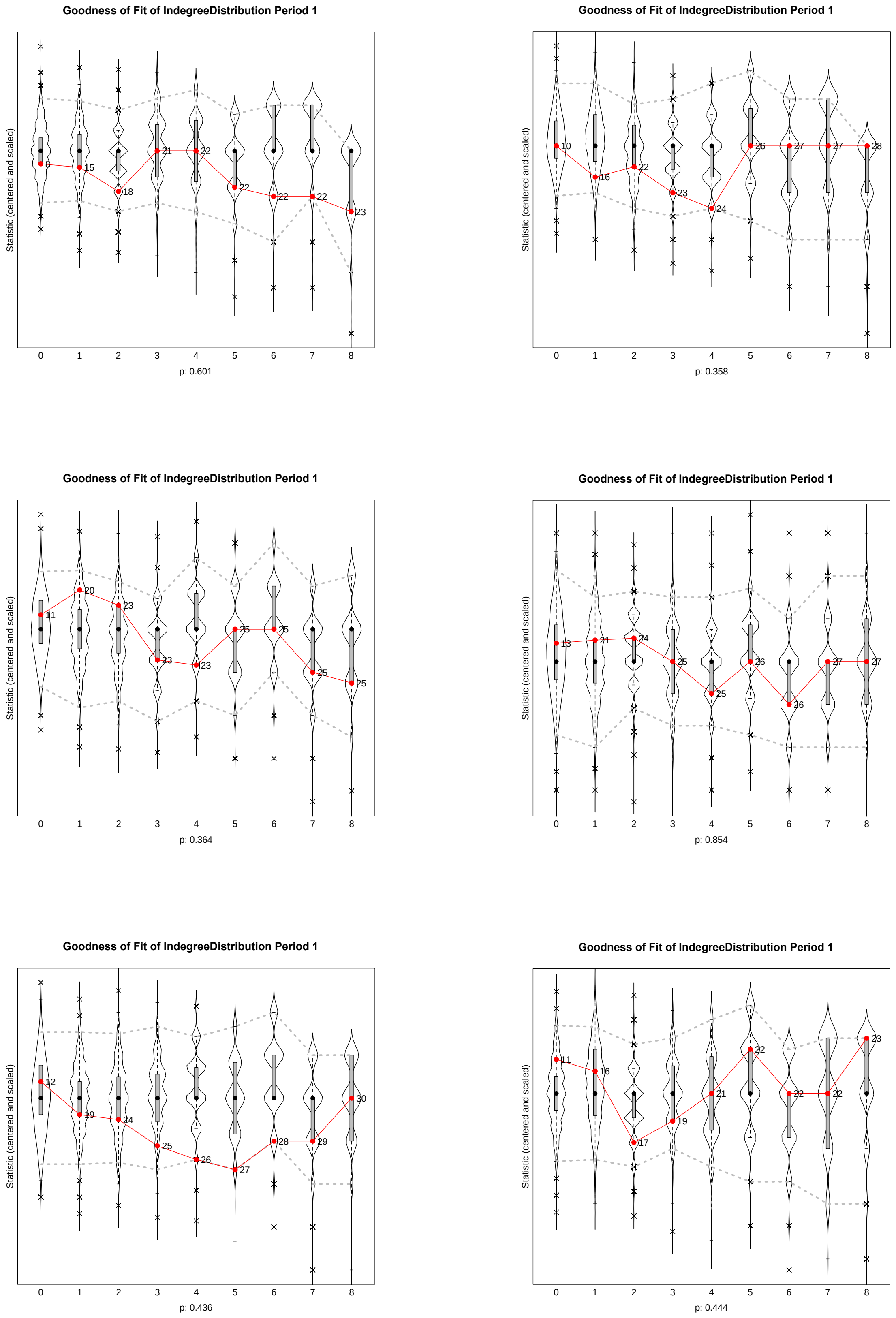

Figure S1. Goodness of fit results of auxiliary statistics (indegree distribution).

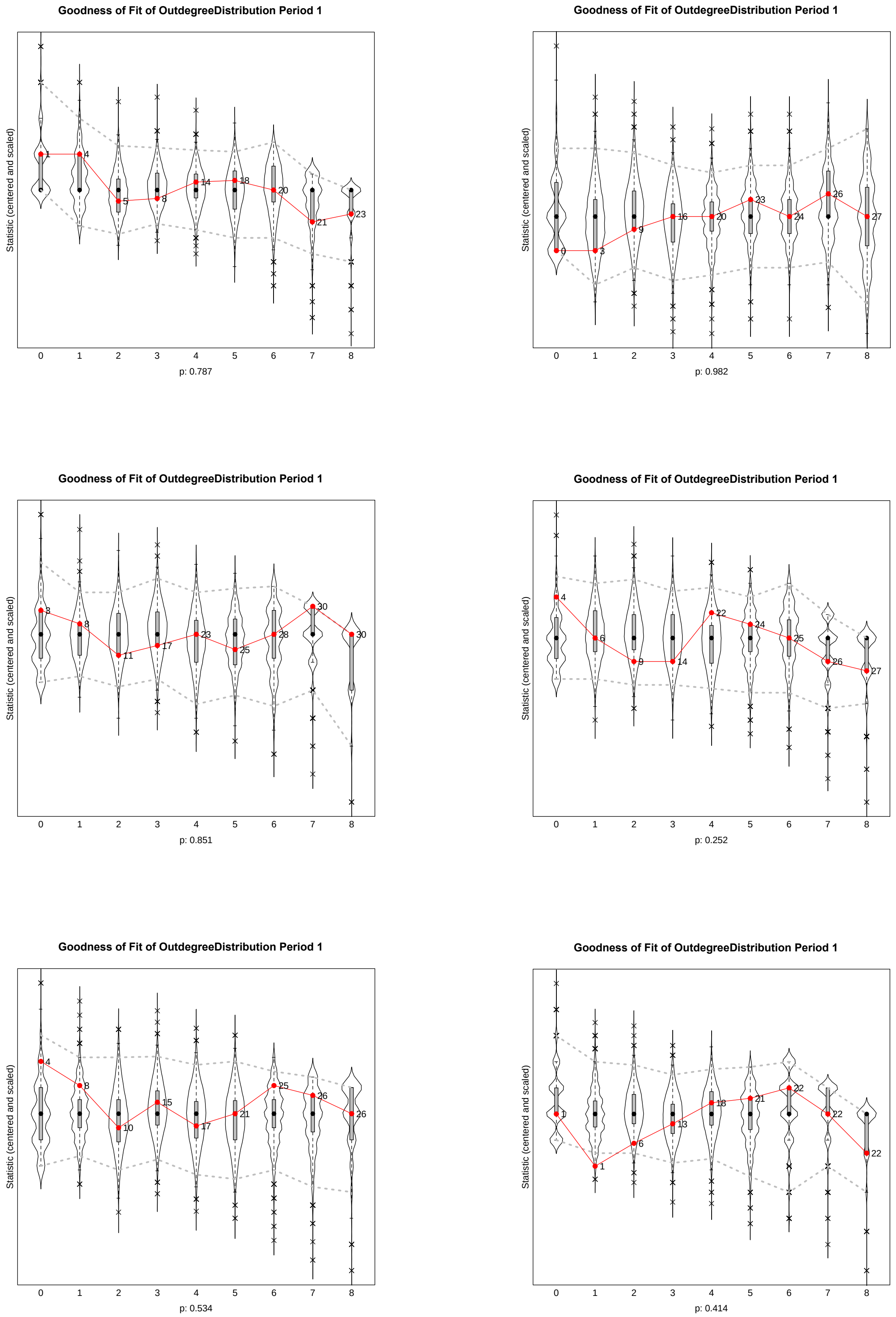

Figure S2. Goodness of fit results of auxiliary statistics (outdegree distribution).

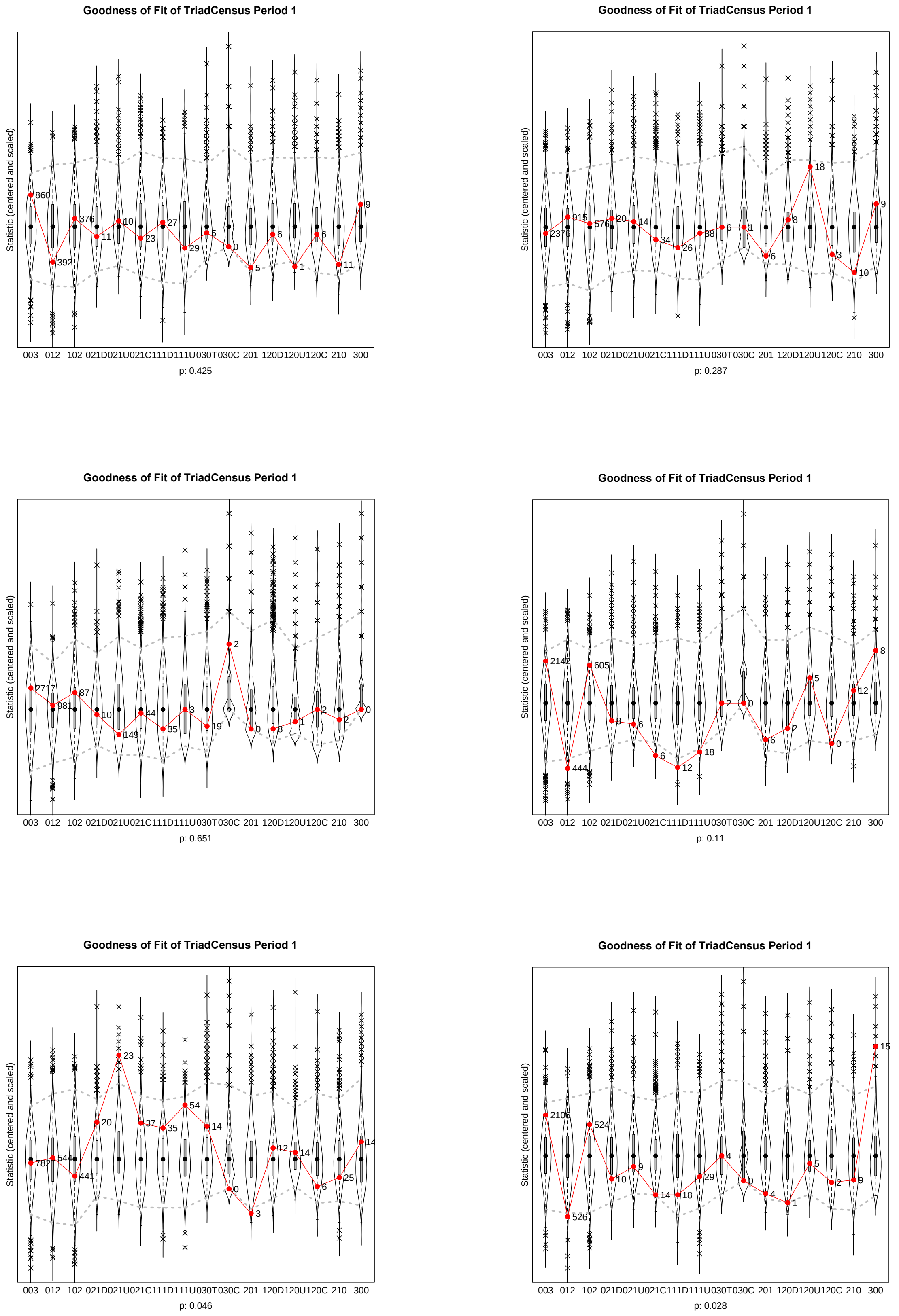

Figure S3. Goodness of fit results of auxiliary statistics (triad census).

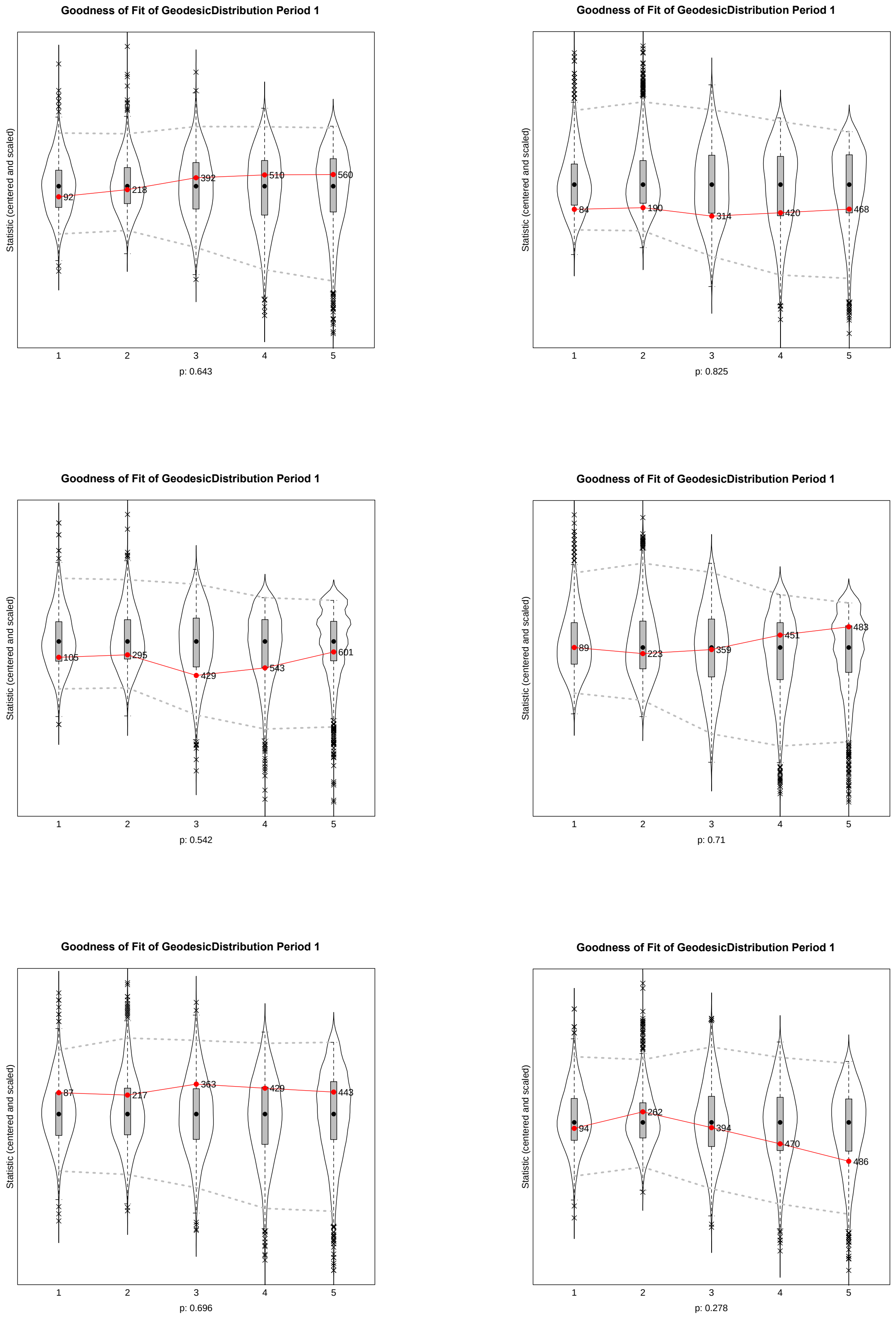

Figure S4. Goodness of fit results of auxiliary statistics (geodesic distance).

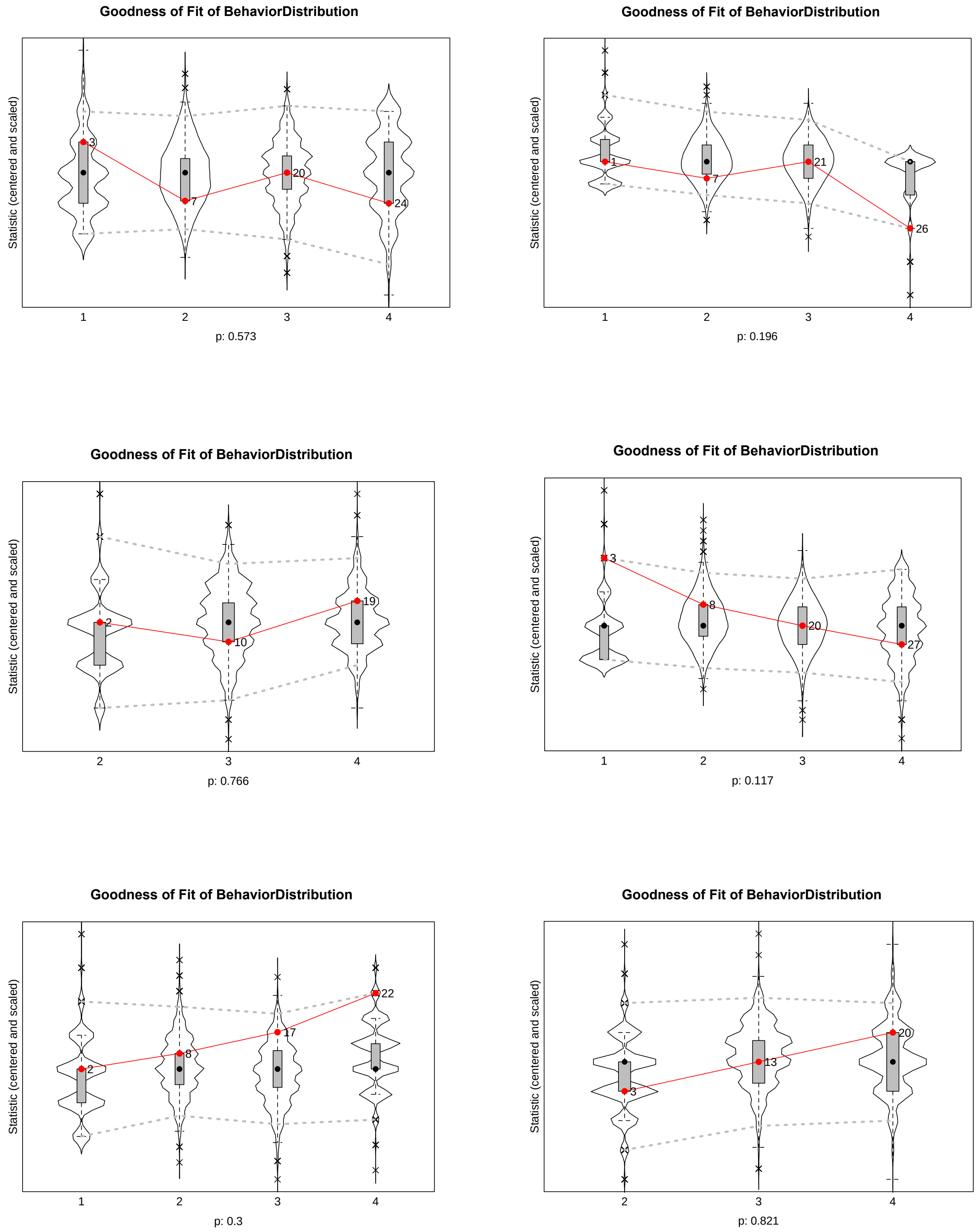

Figure S5. Goodness of fit results of auxiliary statistics (behavioral academic engagement).

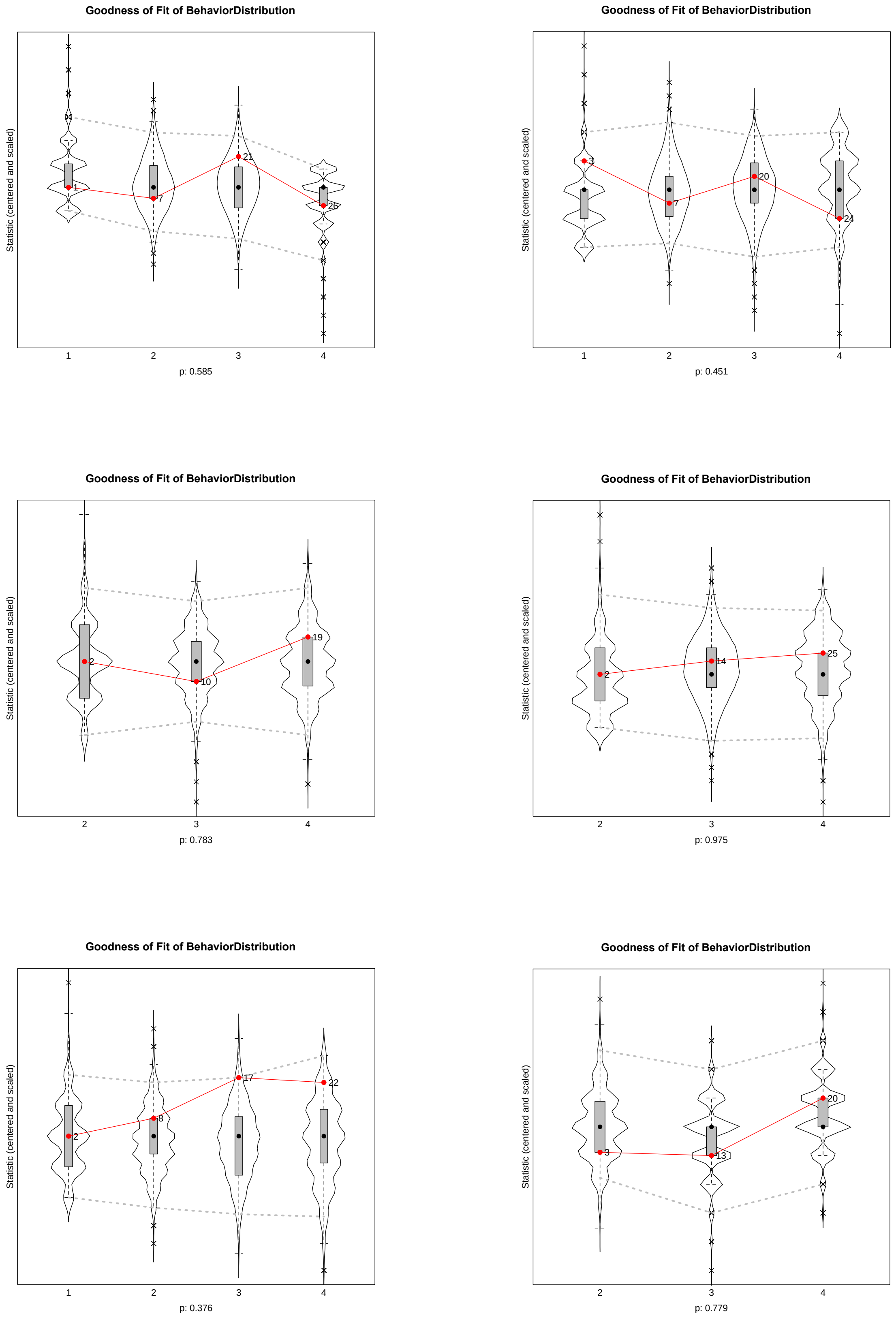

Figure S6. Goodness of fit results of auxiliary statistics (emotional academic engagement).

Appendix 2

Table S1. *Description of basic parameters used in the SAOM model*

Effects	Conceptual description	Graphical representation
Network structural effects		
Outdegree	Basic tendency to form social ties	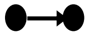
Reciprocity	Tendency to form reciprocated social ties	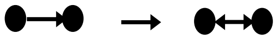
Transitive triplets	Tendency to form social ties to friends of friends	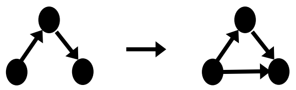
Transitive reciprocated triplets	Tendency to form or remain mutual friends when part of transitive triplets	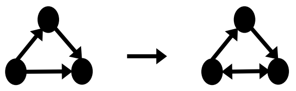
Three-cycles	Tendency to form three-cycles, which is the simplest form of generalized exchange and is opposed to hierarchy	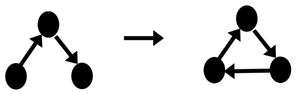
Indegree popularity	Tendency of actors to send social ties to those who are popular in the network	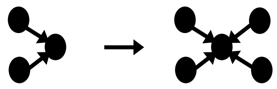

Outdegree activity	Tendency of actors who nominate many others as social contacts to nominate more social ties	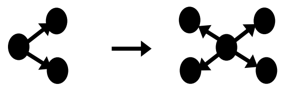
Network selection effects		
Alter effect	Tendency to receive social ties based on the individual's mean level on the attribute	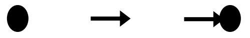
Ego effect	Tendency to select social ties based on the individual's mean level on the attribute	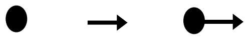
Similar selection	Tendency to select each other based on similarity between the individual and social ties in the attribute	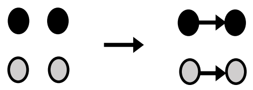
Behavior dynamic effects		
Linear shape	Tendency toward high or low values on the attribute	
Quadratic shape	Tendency of self-reinforcing (positive estimate) or self-correcting (negative estimate) effect	
Average similarity	Tendency to become more similar in the attribute over time: social tie's attribute predicts changes in the individual's attribute; social influence	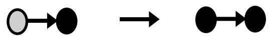

Note. Information was derived from RSiena manual [59].

Appendix 3

Table S2. *Ego-alter selection table: Selection of friends (alters) on early adolescents' (egos') behavioral academic engagement*

Individual's B.E.	Friends' behavioral academic engagement (B.E.)				
	1	2	3	4	5
1	-0.01	-0.16	-0.31	-0.46	-0.61
2	**0.07**	**0.02**	-0.13	-0.28	-0.43
3	**0.15**	**0.10**	**0.04**	-0.11	-0.26
4	**0.23**	**0.18**	**0.12**	**0.07**	-0.08
5	**0.32**	**0.26**	**0.21**	**0.15**	**0.10**

Note. Numbers in the table reflect the strength of attraction for adolescents based on their levels of behavioral academic engagement (column are dependent on rows). The values on the lower behavioral engagement scores (i.e., 1–3) indicated that individuals were more likely to select as friends who are less behaviorally engaged peers.
The values on the diagonal indicate the likelihood that individuals will select as friends who have the same levels of behavioral engagement. Comparing the values between rows and columns indicates that friend selection occurs between similarly scoring individuals, and these effects become stronger for the higher behavioral engagement scores.

Table S3. *Ego-alter selection table: Selection of helpers (alters) on early adolescents' (egos') behavioral academic engagement.*

Individual's B.E.	Helpers' behavioral academic engagement (B.E.)				
	1	2	3	4	5
1	-0.57	-0.37	-0.18	0.02	**0.21**
2	-0.60	-0.30	-0.11	**0.09**	**0.28**
3	-0.63	-0.33	-0.04	**0.16**	**0.35**
4	-0.66	-0.36	-0.07	**0.23**	**0.42**
5	-0.69	-0.39	-0.10	**0.20**	**0.50**

Note. Numbers in the table reflect the strength of attraction for adolescents based on their levels of behavioral academic engagement (column are dependent on rows). The values on the higher behavioral engagement scores (i.e., 4, 5) indicated that individuals were more likely to select as helpers who are highly behaviorally engaged peers. Comparing the values between rows and columns indicates that these effects become stronger for the highest behavioral academic engagement scores: Youth with the highest behavioral academic engagement score (i.e., 5) were more likely to be selected as helpers than were youth with the lowest academic engagement score (i.e., 1–3).

Table S4. *Ego-alter influence table: Influence of friends (alters) on early adolescents' (egos') behavioral and emotional academic engagement.*

	Individual's behavioral academic engagement (B.E.)				
Average B.E. of friends	1	2	3	4	5
1	**0.05**	-0.02	-0.39	-1.06	-2.03
2	-0.40	**0.43**	0.06	-0.61	-1.58
3	-0.86	-0.02	**0.52**	-0.15	-1.12
4	-1.31	-0.47	0.06	**0.30**	-0.70
5	-1.76	-0.93	-0.39	-0.15	**-0.22**
	Individual's emotional academic engagement (E.E.)				
Average E.E of friends	1	2	3	4	5
1	**0.62**	0.32	-0.25	-1.12	-2.26
2	0.26	**0.68**	0.10	-0.76	-1.91
3	-0.09	0.32	**0.45**	-0.41	-1.56
4	-0.44	-0.03	0.10	**-0.05**	-1.20
5	-0.80	-0.38	-0.25	-0.41	**-0.85**

Note. Numbers in the table reflect the strength of the social influence on adolescents to change their engagement based on friends' average levels of engagement (columns are dependent on rows). The values in the cells in these tables can be transformed to odds by taking the exponential function (exp.(βk)). The maximum values on the diagonal indicate that for each average friend engagement score, adolescents prefer to have similar levels of engagement of their friends. These effects were stronger for lower scores (i.e., 2–3) than higher scores (i.e., 4–5): Youth with lower engagement scores were more likely to develop lower engagement when they had friends with lower scores than were youth with higher engagement score.

Table S5. *Ego-alter influence table: Influence of helpers(alters) on early adolescents' (egos') behavioral and emotional academic engagement.*

	Individual's behavioral academic engagement (B.E.)				
Average B.E. of helpers	1	2	3	4	5
1	**0.34**	0.19	-0.46	-1.63	-3.31
2	-0.29	**0.83**	0.17	-1.00	-2.68
3	-0.92	0.19	**0.80**	-0.37	-2.05
4	-1.55	-0.44	0.17	**0.26**	-1.42
5	-2.19	-1.07	-0.46	-0.37	**-0.78**
	Individual's emotional academic engagement (E.E.)				
Average E.E of helpers	1	2	3	4	5
1	**0.73**	0.54	-0.07	-1.11	-2.59
2	0.46	**0.81**	0.20	-0.85	-2.32
3	0.19	0.54	**0.46**	-0.58	-2.05
4	-0.08	0.27	0.20	**-0.31**	-1.78
5	-0.34	0.01	-0.07	-0.58	**-1.52**

Note. Numbers in the table reflect the strength of the social influence on adolescents to change their engagement based on helpers' average levels of engagement (columns are dependent on rows). The values in the cells in these tables can be transformed to odds by taking the exponential function (exp.(βk)). The maximum values on the diagonal indicate that for each average helper engagement score, adolescents prefer to have similar levels of engagement of their helpers. These effects were stronger for lower scores (i.e., 2–3) than higher scores (i.e., 4–5).
